# Multidimensional Sleep Health Is Associated with Cardiovascular Disease Prevalence and Cardiometabolic Health in US Adults

**DOI:** 10.3390/ijerph191710749

**Published:** 2022-08-29

**Authors:** Nour Makarem, Carmela Alcantara, Sydney Musick, Odayme Quesada, Dorothy D. Sears, Ziyu Chen, Parisa Tehranifar

**Affiliations:** 1 Department of Epidemiology, Mailman School of Public Heath, Columbia University Irving Medical Center, New York, NY 10032, USA; 2 School of Social Work, Columbia University, New York, NY 10027, USA; 3 Women’s Heart Center, The Christ Hospital Heart and Vascular Institute, Cincinnati, OH 45219, USA; 4 College of Health Solutions, Arizona State University, Phoenix, AZ 85004, USA; 5 Department of Medicine, University of California San Diego, San Diego, CA 92093, USA; 6 Center for Circadian Biology, University of California San Diego, San Diego, CA 92093, USA

**Keywords:** multidimensional sleep health, cardiovascular disease, hypertension, type 2 diabetes, obesity, central adiposity

## Abstract

Individual sleep dimensions have been linked to cardiovascular disease (CVD) risk and cardiometabolic health (CMH), but sleep health is multifaceted. We investigated associations of a multidimensional sleep health (MDSH) score, enabling the assessment of sleep health gradients, with CVD and CMH. Participants were 4555 adults aged ≥20 years from the 2017–2018 National Health and Nutrition Examination Survey. A MDSH score, capturing poor, moderate, and ideal sleep was computed from self-reported sleep duration, sleep regularity, difficulty falling asleep, symptoms of sleep disorders, and daytime sleepiness. Survey-weighted multivariable linear and logistic models examined associations of MDSH with CVD and CMH. Ideal and moderate vs. poor MDSH were related to lower odds of hypertension (62% and 41%), obesity (73% and 56%), and central adiposity (68% and 55%), respectively; a statistically significant linear trend was observed across gradients of MDSH (*p*-trend < 0.001). Ideal vs. moderate/poor MDSH was associated with 32% and 40% lower odds of prevalent CVD and type 2 diabetes, respectively. More favorable MDSH was associated with lower blood pressure, BMI, waist circumference, and fasting glucose. In sex-stratified analyses, ideal vs. moderate/poor MDSH was associated with lower CVD odds and blood pressure in women only. The MDSH framework may be more than just the sum of its parts and could better capture information regarding CVD risk.

## 1. Introduction

Sleep is an important contributor to cardiometabolic health (CMH) [1]. Poor sleep is ubiquitous with 50–70 million US adults having a sleep disorder or insufficient sleep [2]. Furthermore, approximately 35% of US adults are short sleepers (<7 h/night), up to 20% report excessive daytime sleepiness, <50% report having a good night of sleep every night, and ~65% have variable sleep duration and timing patterns [3,4,5,6,7,8]. Importantly, sleep disorders, such as insomnia and obstructive sleep apnea, have been linked to other adverse sleep characteristics, such as short sleep duration and daytime sleepiness, suggesting that multiple unhealthy sleep characteristics may occur concurrently, further augmenting risk for cardiometabolic disease [5,9,10,11].

Sleep may play a role in cardiovascular disease (CVD) etiology via its influence on cardiometabolic risk factors [1,12]. Indeed, several sleep characteristics have been independently linked to CMH in observational and experimental studies [1]. A 2016 American Heart Association (AHA) scientific statement on sleep and CMH concluded that sleep duration, mainly short and poor quality sleep, and sleep disorders are associated with higher risk for obesity, type 2 diabetes (T2D), and hypertension (HTN) [1]. Recently, novel aspects of sleep such as sleep irregularity, namely greater variability in timing and duration of sleep, have also been linked to adverse cardiometabolic outcomes and CVD risk [7,8]. However, sleep health is increasingly recognized as a multidimensional construct akin to diet [13,14]. Just as diet is not consumed as a single food resulting in a focus on promoting healthy dietary patterns for CVD risk reduction, sleep dimensions also do not occur in isolation. Therefore, studies that focus on a single sleep dimension may provide incomplete conclusions about the role of sleep in preserving CMH and lowering CVD risk.

The adoption of a multidimensional sleep health (MDSH) framework, first introduced by Buysse, acknowledges that sleep dimensions are distinct but often interrelated, and that sleep health is not the mere absence of sleep disorders but rather the adoption of achievable positive sleep habits that promote disease-free survival and healthy aging [13,14]. Importantly, a MDSH approach recognizes the existence of gradients of healthy sleep, contains actionable targets, can be readily measured and monitored over time, and can be applicable to individual and population-level approaches for CMH promotion [13,14,15].

MDSH has been linked to lower risk of CVD mortality in elderly adults [16] and to lower heart disease prevalence in midlife adults [17], but associations with both prevalent CVD and cardiometabolic risk factors that underlie the continuum of chronic disease and predispose to CVD have not been previously evaluated in a nationally representative cohort of US adults. Further, relations of MDSH scores, that could be easily ascertained in a clinic or public health setting, with odds of having obesity, T2D, and HTN, the leading contributors to CVD morbidity and mortality, have not been previously evaluated. To address these knowledge gaps, we conducted a cross-sectional analysis using the National Health and Nutrition Examination Survey (NHANES 2017–2018) to investigate associations of MDSH scores with CVD and cardiometabolic outcomes. We hypothesized that having more favorable MDSH would be related to lower body mass index (BMI), waist circumference (WC), fasting glucose, and blood pressure (BP), and to lower odds of having CVD, HTN, obesity, central adiposity, and T2D.

## 2. Materials and Methods

### 2.1. Study Population

Participants were adult men and women from the 2017–2018 NHANES, which was conducted by the National Center for Health Statistics of the US Centers for Disease Control and Prevention [18]. NHANES is nationally representative, as study samples are identified through a complex, stratified, multistage probability sampling design (counties, segments, households, and individuals) to ensure external validity [18]. Participants completed in-home interviews to provide socio-demographic, lifestyle, and medical history data. They subsequently visited a mobile examination center where they responded to additional questionnaires, underwent a medical exam, and provided a blood sample. NHANES maintains high standards to ensure minimal non-sampling and measurement error during survey planning, data collection, and data processing [18]. For the present analysis, participants were excluded if they were aged <20 y, were pregnant, and if they had missing data on the sleep dimensions that were used to compute the MDSH score. A total of 4555 participants were included in our final analytic dataset. NHANES is approved by the National Center for Health Statistics Research Ethics Review Board, and all participants included in the present analysis provided written informed consent [18].

### 2.2. Assessment of Sleep

Sleep health information was self-reported on questionnaires [18]. Participants reported the number of hours they usually sleep and about the times they usually fall asleep and wake up on weekdays/workdays and on weekends/non-workdays. A weighted average (5/7 for weekdays and 2/7 for weekends) was computed to ascertain mean habitual sleep duration and the average sleep midpoint. To evaluate sleep pattern regularity, the absolute value of the difference between weekday vs. weekend sleep duration and sleep timing was computed. Participants reported the frequency with which they snore and snort (or stop breathing during their sleep) as never, rarely (1–2 nights/wk), occasionally (3–4 nights/wk), and frequently (5+ nights/wk). They were also asked whether or not they ever told a doctor that they had trouble sleeping (Yes vs. No), and how often they felt excessive daytime sleepiness: never, rarely (once/month), sometimes (2–4 times/month), often (5–15 times/month), and almost always (16–30 times/month).

### 2.3. Operationalization of the Multidimensional Sleep Health Score

The MDSH score was based on the aforementioned self-reported sleep health measures that were available in the 2017–2018 NHANES dataset and included the following dimensions: (1) sleep duration, (2) sleep regularity, (3) sleep latency, (4) symptoms of disordered sleep, and (5) daytime sleepiness (alertness). Participants received a score of 1 or 0, representing optimal or suboptimal responses for each dimension, as follows:

***Sleep duration*:** Participants received a score of 1 if habitual sleep duration was ≥7 h and <9 h, consistent with the definition of sufficient sleep in the AHA scientific statement on sleep and CMH [1]. A score of 0 was assigned for sleep duration <7 h and ≥9 h (short and long sleep).

***Sleep regularity:*** The weekday-weekend difference in sleep midpoint (i.e., sleep timing) and sleep duration was computed. Those with weekday-weekend differences in sleep duration and sleep timing <2 h were assigned a score of 1 to indicate more regular sleep patterns. Those with weekday-weekend differences in sleep duration and/or sleep timing ≥2 h were assigned a score of 0 to indicate variable sleep patterns, given that the strongest associations between social jetlag (i.e., weekday-weekend differences in sleep timing) and cardiometabolic risk in previous research have been observed for social jetlag ≥2 h [19,20].

***Sleep latency:*** Those who responded that they have never told their doctor that they have trouble falling asleep were assigned a score of 1, while those who indicated that they have trouble falling asleep were considered to have poor sleep latency (difficulty initiating sleep) and were assigned a score of 0.

***Symptoms of sleep disorders:*** Participants who reported never or rarely snoring, snorting, or having difficulty breathing during sleep were considered to have no significant symptoms of sleep disorders and were thus assigned a score of 1. Those who reported frequently or often having these symptoms were assigned a score of 0.

***Daytime sleepiness/Alertness:*** Those who reported never or rarely having excessive daytime sleepiness were assigned a score of 1, while those who reported having excessive daytime sleepiness often or frequently, indicating low alertness, received a score of 0.

The individual sleep dimensions’ scores were summed to create an overall MDSH score ranging from 0–5, such that higher scores were indicative of more favorable sleep health. Further, the score was categorized as 0–1, 2–3, and 4–5 that captured poor, moderate, and ideal sleep health, respectively.

### 2.4. Ascertainment of Cardiovascular Disease

Participants reported their health conditions and medical history on the medical conditions questionnaire. Those who indicated that they had ever been told that they have one or more of the following: heart failure, coronary heart disease, angina/angina pectoris, heart attack, or stroke, were considered to have CVD. There were 530 cases of prevalent CVD.

### 2.5. Assessment of Cardiometabolic Risk Factors

During the mobile examination center medical exam, anthropometric measures were collected by trained health technicians. WC was measured above the iliac crest using a measuring tape, and the value was rounded to the nearest 0.1 cm. Height and weight were measured using a stadiometer and a digital weight scale, respectively, and used to calculate BMI (kg/m^2^). Plasma glucose concentrations (mg/dL) were measured from fasting blood specimens. Four BP measures were obtained using the right arm after resting in a seated position for 5 min, and the average of the first three measurements was used, given the large number of missing values for the fourth measurement. Participants self-reported HTN and T2D medication use on questionnaires.

Two definitions were used to ascertain HTN. Definition 1 was based on the 2017 ACC/AHA guidelines, such that individuals who had systolic BP (SBP) ≥ 130 mmHg or diastolic BP (DBP) ≥ 80 mmHg or took any prescribed drugs to control BP were categorized as having HTN [21]. Definition 2 was based on the Seventh Report of the Joint National Committee on Prevention, Detection, Evaluation, and Treatment of High Blood Pressure (JNC 7) guidelines, such that individuals who had SBP ≥ 140 mmHg or DBP ≥ 90 mmHg or took any prescribed drugs to control BP were categorized as having HTN [22]. T2D was defined as fasting glucose ≥126 mg/dL, or self-reported use of oral hypoglycemic medication and/or insulin, consistent with AHA and American Diabetes Association definitions [23,24]. Obesity and central adiposity were defined by BMI ≥ 30 kg/m^2^ and WC > 102 cm for men and > 88 cm for women, respectively [25,26].

### 2.6. Statistical Analysis

Participants’ socio-demographic, sleep, and cardiometabolic risk profile characteristics were described using mean ± SD for continuous variables and frequencies for categorical variables. Survey-weighted linear regression models were used to evaluate associations of the MDSH score (categorical) and component scores (categorical) with the following cardiometabolic risk factors (continuous): BMI (kg/m^2^), WC (cm), fasting glucose (mg/dL), SBP (mmHg), and DBP (mmHg). Linear models were also used to examine individual sleep health dimensions, available on the continuous scale (sleep duration and weekday-weekend differences in sleep timing and duration), in relation to aforementioned cardiometabolic risk factors.

Survey-weighted logistic regression models were used to evaluate the MDSH score categories (ideal vs. moderate/poor and ideal vs. moderate vs. poor) and component scores in relation to odds of prevalent CVD, HTN, obesity, central adiposity, and T2D. The test for linear contrast was used to compute *p*-trend values for the detection of a linear trend across categories of the MDSH score. All models were adjusted for the following a priori selected covariates: age (years), sex (male, female), race/ethnicity (non-Hispanic White, non-Hispanic Black, Hispanic, non-Hispanic Asian, other race/more than one race), marital status (married/living with partner, single/divorced/widowed), education (≥some college, <college), and medication use (HTN or T2D medications). In exploratory analyses, we evaluated sex differences in all associations, and sex-stratified results were reported when significant. A *p*-value < 0.05 was considered significant, and SAS version 9.4 (Cary, NC, USA) was used to conduct all analyses.

## 3. Results

### 3.1. Characteristics of the Study Population

The mean age was 49 ± 18 years (range: 20–80 years), and 55% of participants were aged ≥45 years (Table 1). Overall, half were female, 63% had an education level ≥some college education, and 64% were married or living with a partner. The racial and ethnic distribution was as follows: 63% non-Hispanic White, 11% Non-Hispanic Black, 16% Hispanic, and 6% Non-Hispanic Asian. In terms of their sleep health, 41%, 50%, and 9% had an ideal, moderate, and poor MDSH score, respectively. About a quarter of participants reported sleep duration <7 h/night and 18% were long sleepers (≥9 h), 40% had a variable sleep pattern based on weekday-weekend differences in their sleep duration and timing, 30% reported having difficulty falling asleep, 27% reported often/always having excessive daytime sleepiness, and 48% reported snoring and/or snorting ≥3 nights/week.

### 3.2. Multidimensional Sleep Health and Cardiometabolic Risk Factors

***Body Mass Index:*** Ideal vs. moderate/poor MDSH was associated with 2.53 kg/m^2^ lower BMI (*p <* 0.001) (Table 2). When comparing three categories of the MDSH score, participants with ideal and moderate vs. poor MDSH had significantly lower BMI (β(95%CI): −5.34 (−6.50, −4.17) and −3.26 (−4.51, −2.01), respectively). Those who reported never/rarely snoring and/or snorting, never/rarely/sometimes having excessive daytime sleepiness, and no difficulty falling asleep had lower BMI (*p <* 0.001). Although the sleep duration score was not related to BMI, longer sleep duration on the continuous scale was associated with lower BMI (β(95%CI): −0.28 (−0.52, −0.04)), and those who slept <7 h vs. ≥7 h had higher BMI (β(95%CI): 0.89 (0.06–1.72)). Although the sleep variability component score was not related to BMI, greater weekday-weekend differences in sleep duration were related to higher BMI (β(95%CI): 0.29 (0.07, 0.51)), but no association was observed for weekday-weekend differences in sleep timing.

***Waist Circumference:*** Ideal vs. moderate/poor MDSH was associated with 6.06 cm lower WC (*p <* 0.001) (Table 2). Participants with ideal and moderate compared to poor MDSH had lower WC (β(95%CI): −13.32 (−15.42, −11.23) and −8.44 (−10.81,−6.07), respectively). When component scores were evaluated, those with favorable scores for symptoms of sleep disorders, sleep latency, and alertness had lower WC. Greater weekday-weekend differences in sleep duration were associated with higher WC (β(95%CI):0.53 (0.02–1.04)).

***Fasting Glucose:*** Ideal vs. moderate/poor MDSH was associated with 2.76 mg/dL (95%CI: −4.98,−0.54) lower fasting glucose (Table 2). Although ideal vs. poor MDSH was related to significantly lower fasting glucose (β(95%CI): −6.40 (−11.24, −1.56)), this relation was not significant when comparing moderate vs. poor MDSH. No statistically significant associations were observed for component scores. However, having sleep duration <6 h vs. ≥6 h was related to higher fasting glucose levels (β(95%CI):8.16 (0.66, 15.65)).

***Systolic Blood Pressure:*** Ideal vs. moderate/poor MDSH was associated with 1.97 mmHg lower SBP (*p =* 0.026) (Table 2). Participants with ideal and moderate vs. poor MDSH also had lower SBP (β(95%CI): −4.22 (−6.95, −1.49) and −2.60 (−5.06, −0.14)), respectively). When component scores were evaluated, never/rarely vs. occasionally/frequently snoring and/or snorting was related to lower SBP.

***Diastolic Blood Pressure:*** Ideal vs. moderate/poor MDSH was associated with 1.16 mmHg (95%CI: −1.82,−0.50) lower DBP (Table 2). Further, participants with ideal and moderate vs. poor MDSH had significantly lower DBP (β(95%CI): −3.42 (−5.38, −1.47) and −2.61 (−4.33, −0.90), respectively). Those with favorable component scores for symptoms of sleep disorders and sleep latency had lower DBP (*p <* 0.001). Those with favorable sleep regularity scores had 1.26 mmHg lower DBP but this relation was not statistically significant (*p =* 0.052). Although the sleep duration score was not related to DBP, each 1-h increase in habitual sleep duration was associated with lower DBP (β(95%CI): −0.38 (−0.71, −0.04), while each 1-h increase in weekday-weekend differences in sleep duration, indicative of greater sleep variability, was related to higher DBP (β(95%CI):0.66 (0.01, 1.32)).

### 3.3. Multidimensional Sleep Health and Prevalent Cardiometabolic Outcomes

**Figure 1:**
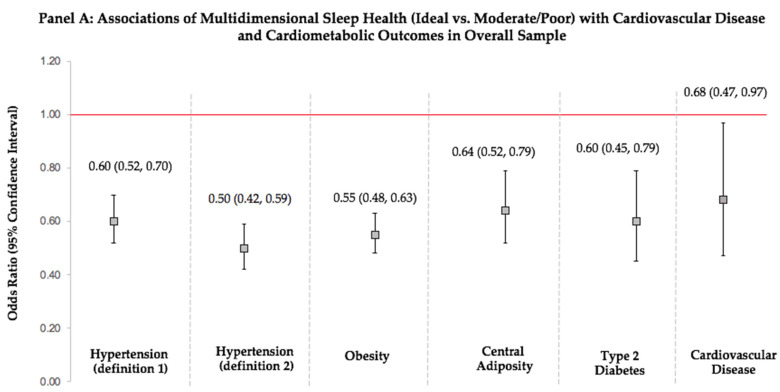

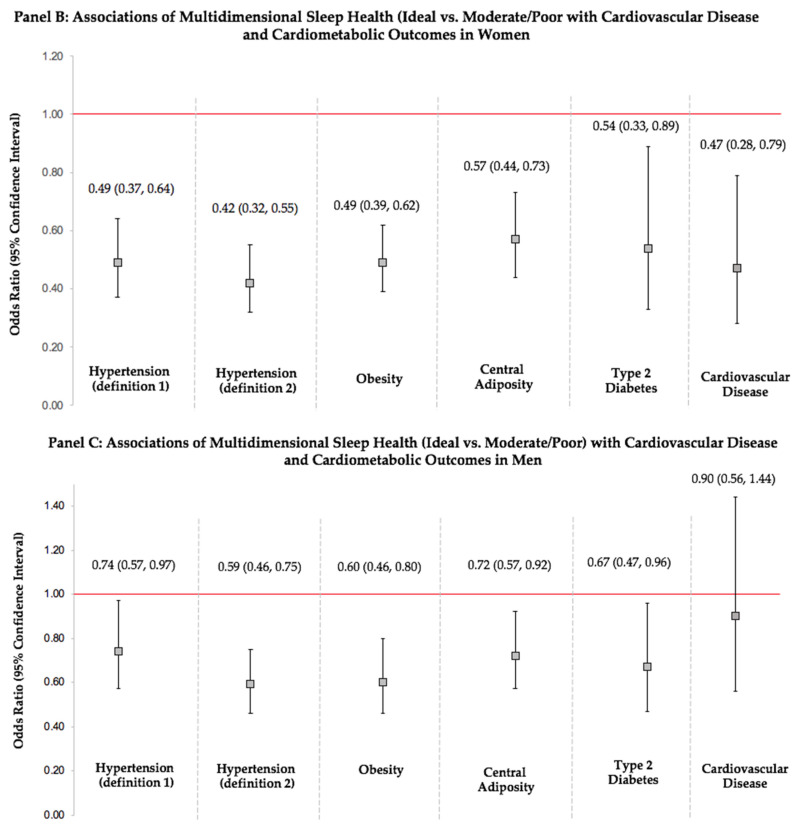
Associations of Multidimensional Sleep Health with Prevalent Cardiovascular Disease and Cardiometabolic Outcomes in the Overall Sample and By Sex (n = 4555)**. Caption.** Associations of ideal vs. moderate/poor multidimensional sleep health with prevalent cardiovascular disease and cardiometabolic outcomes from logistic regression models. All models were adjusted for age, sex, race/ethnicity, education, and marital status. Obesity is defined as a BMI ≥ 30 kg/m^2^; central adiposity is defined as a waist circumference > 88 cm in women and > 102 cm in men; and type 2 diabetes is defined as fasting glucose ≥126 mg/dL or type 2 diabetes medication use. Hypertension definition 1 (2017 ACC/AHA definition) is SBP/DBP ≥ 130/80 mmHg or hypertension medication use, and hypertension definition 2 (JNC 7 definition) is SBP/DBP ≥ 140/90 mmHg or hypertension medication use. The CVD composite outcome includes heart failure, coronary heart disease, angina/angina pectoris, heart attack, or stroke. Panel A displays associations in the overall sample, and panels B and C display associations in women and men, respectively.

***Obesity:*** Ideal vs. moderate/poor MDSH was related to 45% lower odds of prevalent obesity (Figure 1, Panel A). Ideal and moderate vs. poor MDSH were associated with 73% and 56% lower odds for obesity, respectively, and a statistically significant linear trend across the gradients of MDSH was observed (*p*-trend < 0.001) (Table 3). Those with favorable scores for symptoms of sleep disorders, sleep latency, and daytime sleepiness (alertness) had 58%, 43%, and 43% lower odds of obesity, respectively.

***Central Adiposity:*** Ideal vs. moderate/poor MDSH was related to 36% lower odds of central adiposity (Figure 1, Panel A). Those with ideal and moderate vs. poor MDSH had 68% and 55% lower odds of central adiposity, respectively (*p*-trend < 0.001) (Table 3). Among score components, those with favorable scores for symptoms of sleep disorders, sleep latency, and daytime sleepiness (alertness) had 55%, 32%, and 39% lower odds of having an at-risk WC, respectively.

***Type 2 Diabetes:*** Ideal vs. moderate/poor MDSH was related to 40% lower odds of T2D (Figure 1, Panel A). However, associations were not statistically significant when those with ideal and moderate MDSH were compared to those with poor MDSH (Table 3). Favorable scores for symptoms of sleep disorders, sleep latency, and daytime sleepiness (alertness) were related to 37%, 35%, and 46% lower odds of T2D, respectively.

***Hypertension:*** Ideal vs. moderate/poor MDSH was related to 40% and 50% lower odds of prevalent HTN based on the 2017 ACC/AHA and JNC 7 definitions, respectively (Figure 1, Panel A). Further, ideal and moderate vs. poor MDSH were associated with 62% and 41% lower odds of HTN, based on the 2017 ACC/AHA definition and with 74% and 53% lower odds of HTN, based on the JNC 7 definition (Table 3). A statistically significant linear trend across the gradients of MDSH was observed (*p*-trend < 0.001), highlighting the presence of a dose-response relationship. When component scores were evaluated, those with favorable scores for symptoms of sleep disorders, sleep latency, and daytime sleepiness (alertness) had lower odds of HTN. A favorable sleep duration score was additionally related to 33% lower odds of HTN defined by the JNC 7 criteria.

### 3.4. Multidimensional Sleep Health and Prevalent Cardiovascular Disease

Ideal vs. moderate/poor MDSH was related to 32% lower odds of prevalent CVD (Figure 1, Panel A), but no statistically significant association was observed when ideal and moderate sleep health were compared to poor sleep health. Favorable sleep duration (34%), sleep latency (41%), and daytime sleepiness (alertness) (52%) scores were related to lower odds of prevalent CVD (Table 3). However, a favorable sleep variability score was associated with higher odds of prevalent CVD.

### 3.5. Sex Differences in Associations of Multidimensional Sleep Health with Cardiovascular Disease and Cardiometabolic Health

In exploratory analyses stratified by sex, there were no sex differences in associations observed for DBP, BMI, WC, and fasting glucose. However, ideal vs. moderate/poor MDSH was associated with lower SBP in women only (β(95%CI): −3.18 (−5.88, −0.49)). There were no differences by sex in logistic models that evaluated MDSH in relation to cardiometabolic outcomes (Figure 1, Panels B and C). However, ideal vs. moderate/poor MDSH was significantly associated with 53% lower odds of prevalent CVD in women only.

## 4. Discussion

To our knowledge, this represents one of the earliest investigations of MDSH in relation to both prevalent CVD and CMH in a nationally representative sample of US adults. Our findings demonstrate that more favorable MDSH is related to having lower BMI, WC, fasting glucose, and BP. We also show that having ideal vs. moderate/poor MDSH is related to having lower odds of prevalent CVD, obesity, central adiposity, T2D, and HTN. When odds of cardiometabolic outcomes were compared across three categories of the MDSH score, we observed a statistically significant linear trend in logistic models for obesity, central adiposity, and HTN, highlighting the presence of a dose-response relationship across the gradients of MDSH. Importantly, while the overall MDSH score was consistently associated with all cardiometabolic outcomes, we did not observe significant relations for all score components. These data underscore the importance of embracing a holistic approach to sleep health and indicate that the MDSH framework may be more than just the sum of its parts and could better capture information regarding CVD risk.

Our results are consistent with the growing literature demonstrating that MDSH is related to favorable health outcomes [14,16,27]. Indeed, in a cohort of ~9000 elderly US men and women, aged 65–99 years, poor self-reported MDSH was associated with a 2-fold higher risk for CVD mortality [16]. In the Midlife in the United States study, having more self-reported sleep problems was linked to 54% higher risk for heart disease in a cross-sectional analysis of 6,820 adults [17]. Similarly, in the UK Biobank Study, every 1 unit increase in a 5-point sleep health score based on self-reported sleep duration, chronotype, insomnia symptoms, snoring, and excessive daytime sleepiness was related to 15% lower risk of heart failure over a median follow-up of 10 years [28]. We uniquely report that associations of MDSH with CVD and BP vary by sex in exploratory analyses, as associations were stronger in women. This finding is also consistent with prior literature demonstrating that women may be more vulnerable to the cardiovascular consequences of poor sleep compared to men [29,30].

There are limited data on MDSH in relation to CMH. Consistent with our findings, in 268 adults from the Midlife in the United States study, better sleep health, assessed using both self-report and actigraphy, was related to ~10% lower odds of overall cardiometabolic morbidity [31]. In 221 women enrolled in the Study of Women’s Health Across the Nation (SWAN) Sleep Study, overall MDSH and the individual metrics were not related to BMI or waist-to-hip ratio in cross-sectional and prospective analyses [32]. This is contradictory to our finding of consistent associations between favorable MDSH and lower odds for obesity and central adiposity. It is possible that the modest sample size in the SWAN study may have resulted in limited power to detect associations. Additionally, the SWAN study relied on objectively assessed measures of sleep duration, midpoint, regularity, and efficiency and self-reported measures of satisfaction and alertness and included predominantly midlife women [32], whereas our definition of MDSH included distinct self-reported sleep constructs and our study sample encompassed a broader age range, included both men and women, and was representative of the US population.

Our results for the individual sleep health metrics are consistent with the literature. For sleep duration, we found that sleeping 7–9 h was related to lower odds for HTN and CVD, while shorter sleep was related to higher BMI and fasting glucose. This is consistent with the 2016 AHA scientific statement on sleep and CMH, which noted that there is strong evidence from observational and experimental studies that short sleep is a risk factor for obesity, T2D, and HTN [1]. We also found that frequent snoring, snorting, or difficulty breathing during sleep, and excessive daytime sleepiness, which can be symptoms of sleep disorders such as sleep apnea, were consistently related to all cardiometabolic outcomes. There is convincing evidence that sleep apnea is related to obesity, T2D, and HTN etiology [1,30,33]. Further, snoring has been linked to poorer cardiovascular health and higher cardiometabolic risk scores that encompass BMI, BP, and fasting glucose [34,35], and in a meta-analysis to 46% higher risk of incident stroke [36]. Difficulty falling asleep (an insomnia complaint) was also consistently related to all study outcomes and has been extensively linked to cardiometabolic risk factors [1]. According to the 2016 AHA scientific statement on sleep, there is convincing evidence that insomnia symptoms are associated with greater risk for obesity, T2D, and HTN [1], and in a meta-analysis insomnia symptoms were related to 45% higher risk of developing or dying from CVD [37].

We found that those with more regular sleep patterns, defined by lower weekday-weekend differences in sleep duration and timing, had lower DBP, and those with greater weekday-weekend differences in sleep duration had higher BMI, WC, and DBP. This is consistent with existing evidence that sleep variability is an emerging risk factor for obesity and HTN [19,20]. Indeed, in the Multi-Ethnic Study of Atherosclerosis (MESA), irregular sleepers had higher rates of obesity and HTN [38], and every 1-h increase in sleep duration and timing SD, representing greater day-to-day variability in these metrics, was associated with up to 27% greater odds of metabolic syndrome [7]. Irregular sleep duration and sleep timing have also been linked to >2-fold higher risk of developing a CVD event [8], which is inconsistent with our finding that less variable sleep is related to higher odds of CVD. The discrepancy in findings for CVD could be explained by the differences in study design, as MESA is a prospective cohort that evaluated day-to-day sleep variability at baseline in relation to CVD incidence. Given that our study is cross-sectional, we cannot establish temporality; thus, it is possible that those with pre-existing chronic conditions such as CVD have less variable sleep patterns. Further, we did not assess day-to-day variability in sleep duration and timing, as these data were only available for weekdays vs. weekends. It is possible that those with CVD have less discrepancy in the timing and duration of their sleep between weekdays and weekends, as they were less likely to be employed compared to their counterparts without CVD (23% vs. 61%) and may therefore have less social pressure.

Potential mechanisms underlying the association of sleep with CMH include the influence of sleep on other health behaviors. Short sleep, poor quality sleep, and insomnia have been associated with higher caloric intake and unhealthy food choices, including lower intake of plant-based foods and higher intakes of added sugar and sodium [39,40]. Daytime sleepiness and short sleep duration have been linked to lower levels of physical activity and lower odds of meeting physical activity guidelines [41,42]. In MESA, sleep apnea has been associated with poorer quality diet and less physical activity [43,44]. Beyond its influence on health behaviors, sleep may contribute to cardiometabolic risk via other psychological and physiological pathways [1,19,20,30,45]. Sleep deprivation and sleep disturbances have been linked to increased sympathetic activity, reduced parasympathetic activity, increased inflammation, and oxidative stress, which collectively lead to dysfunction of the vascular endothelium and could increase BP [30,46]. In addition, short sleep duration and irregular sleep patterns could disrupt circadian rhythmicity and lead to circadian misalignment and metabolic dysfunction, predisposing to obesity, T2D, HTN, and CVD [1,19,20,30]. Sleep disturbances may also increase cardiometabolic risk via their influence on psychological risk factors, as poor sleep health has been linked to depression, stress, and anxiety [30,45]. Finally, it is possible that poor sleep can increase risk for T2D, HTN, and CVD via its influence on BMI. Although we hypothesize that BMI is a partial mediator of these associations, we evaluated the influence of additionally adjusting our linear and logistic models for BMI; statistically significant associations of MDSH with odds for CVD, HTN, and T2D persisted.

Strengths of this study include the large, nationally representative, and racially and ethnically diverse cohort and the high-quality sampling procedures of NHANES, which enhance statistical power and generalizability of study findings and minimize sampling bias. Weight, height, WC, and BP were objectively measured in mobile examination clinics by trained staff, and fasting glucose was ascertained from a blood specimen using gold standard techniques [18]. Limitations include the lack of data on other sleep dimensions that are well-established contributors to CMH, such as sleep quality and satisfaction. In addition, sleep dimensions were self-reported, which may have resulted in measurement error. Nonetheless, subjective measures of sleep health have been consistently shown to be predictive of cardiometabolic risk, over and beyond objective measures [13,14]. Furthermore, given that an objective assessment of sleep in clinic or public health settings is not always feasible, we believe that the evaluation of a self-reported MDSH score in relation to cardiometabolic outcomes may be clinically relevant.

## 5. Conclusions

Our study uniquely demonstrates that a favorable self-reported MDSH score is associated with lower odds for obesity, central adiposity, T2D, HTN, and CVD. Additional longitudinal studies are warranted to confirm these findings, determine whether associations of MDSH with CMH and CVD vary by life stage, race, and ethnicity, and decipher the role of MDSH in lifetime risk for CVD and extending healthspan. Clinical trials are also needed to evaluate the impact of screening for sleep problems and improving MDSH using behavioral interventions on cardiometabolic outcomes and lowering CVD risk.

## Figures and Tables

**Table 1:** Descriptive Characteristics of the Study Population (n = 4555).

Sociodemographic Factors	%	Sleep Factors *	%	Cardiometabolic Risk Factors	Mean ± SD	Cardiovascular Disease and Cardiometabolic Outcomes	%
**Sex**		Sleep Health Categories *		**Body Mass Index (kg/m^2^)**	29.8 ± 7.4	**Obesity** ^‡^	41.8%
*Women*	51.1%	*Poor (0–1)*	8.5%				
*Men*	48.9%	*Moderate (2–3)*	50.3%				
		*Ideal (4–5)*	41.2%				
**Age Group**		**Sleep Duration** *		**Waist Circumference (cm)**	100.7 ± 17.2	**Central Adiposity** ^‡^	57.1%
20–44 *y*	44.7%	*Short or Long Sleeper*	40.8%				
45–80 *y*	55.3%	*Sufficient Sleeper*	59.2%				
**Nativity**		**Sleep Regularity** *		**Fasting Glucose (mg/dL)**	110.3 ± 37.1	**Type 2 Diabetes** ^‡^	14.7%
*Born in US Mainland*	81.1%	*Variable sleep duration and/or timing*	40.2%				
*Born Outside of US*	18.9%	*Regular sleep duration and/or timing*	59.8%				
**Marital Status**		**Disordered Sleep** *		**Systolic Blood Pressure (mmHg)**	122.8 ± 19.3	**Hypertension (2017 ACC/AHA Definition)** ^†^	43.8%
*Married/Living with Partner*	64.3%	*Frequent snoring/snorting*	47.9%				
*Single/Widowed/Divorced*	35.7%	*Never/Rare snoring/snorting*	52.1%				
**Race/Ethnicity**		**Sleep Latency** *		**Diastolic Blood Pressure (mmHg)**	73.4 ± 12.2	**Hypertension (JNC 7 Definition)** ^†^	31.2%
*NH White*	62.9%	*Difficulty Falling Asleep*	30.1%				
*NH Black*	10.9%	*No Difficulty Falling Asleep*	69.9%				
*NH Asian*	6.0%						
*Hispanic*	15.6%						
*Other Race/Multi-Racial*	4.6%						
**Education**		**Daytime Sleepiness (Alertness)** *		**-**	-	**Cardiovascular Disease** ^§^	11.6%
*Less than College*	37.3%	*Often/Frequent Daytime Sleepiness*	27.3%				
*Some College and Above*	62.7%	*Never/Rare Daytime Sleepiness*	72.7%				

* Multidimensional sleep health scores were computed by assigning a score of 0 or 1 based on self-reported sleep duration, sleep regularity, snoring/snorting, difficulty falling asleep, and daytime sleepiness (alertness). Participants received a score of 1 for sufficient sleep duration (≥7 h and <9 h), regular sleep schedules (weekday-weekend differences in sleep timing midpoint and sleep duration <2 h), never or rarely reporting snoring/snorting, having no difficulty falling asleep, and never or rarely reporting excessive daytime sleepiness. Otherwise, they were assigned a score of 0. Component scores were summed to create the multidimensional sleep health score ranging from 0–5, with higher scores representing more favorable sleep health. Those with scores of 0–1, 2–3, 4–5 were considered to have poor, moderate, and ideal sleep health respectively. ^†^ Hypertension definition 1 (2017 ACC/AHA): SBP/DBP ≥ 130/80 mmHg or hypertension medication use; hypertension definition 2 (JNC 7): SBP/DBP ≥ 140/90 mmHg or hypertension medication use. ^‡^ Obesity is defined as a BMI ≥ 30 kg/m^2^; central adiposity is defined as a waist circumference > 88 cm in women and > 102 cm in men; and type 2 diabetes is defined as fasting glucose ≥126 mg/dL or type 2 diabetes medication use. ^§^ The CVD composite outcome includes heart failure, coronary heart disease, angina/angina pectoris, heart attack, or stroke.

**Table 2:** Multivariable-Adjusted Linear Regression Models for Associations of Multidimensional Sleep Health with Body Mass Index, Waist Circumference, Fasting Glucose, and Blood Pressure (n = 4555) ^†^.

Sleep Health Scores *	Body Mass Index (kg/m^2^)	Waist Circumference (cm)	Fasting Glucose (mg/dL)	Systolic Blood Pressure (mmHg)	Diastolic Blood Pressure (mmHg)
	β (95% CI) *p*-Value	β (95% CI) *p*-Value	β (95% CI) *p*-Value	β (95% CI) *p*-Value	β (95% CI) *p*-Value
MDSH Score *					
**Poor (0–1) (ref)**	ref	ref	ref	ref	ref
**Moderate (2–3)**	−3.26 (−4.51, −2.01) *p* < 0.001	−8.44 (−10.81, −6.07) *p* < 0.001	−4.21 (−9.53, 1.11) *p* = 0.113	−2.60 (−5.06, −0.14) *p* = 0.040	−2.61 (−4.33, −0.90) *p* = 0.005
**Ideal (4–5)**	−5.34 (−6.50, −4.17) *p* < 0.001	−13.32 (−15.42, −11.23) *p* < 0.001	−6.40 (−11.24, −1.56) *p* = 0.013	−4.22 (−6.95, −1.49) *p* = 0.005	−3.42 (−5.38, −1.47) *p* = 0.002
MDSH Score *					
***Ideal* vs. *Moderate/Poor***	−2.53 (−2.98, −2.09) *p <* 0.001	−6.06 (−7.47, −4.65) *p <* 0.001	−2.76 (−4.98, −0.54) *p =* 0.018	−1.97 (−3.67, −0.26) *p =* 0.026	−1.16 (−1.82, −0.50) *p =* 0.002
Sleep Duration Score (*1* vs. *0*) *	−0.29 (−1.06, 0.48) *p =* 0.438	−0.53 (−2.11, 1.05) *p =* 0.487	−3.22 (−6.86, 0.42) *p =* 0.079	−0.34 (−2.00, 1.32) *p =* 0.673	0.49 (−0.52, 1.49) *p =* 0.318
Sleep Regularity Score (*1* vs. *0*) *	−0.29 (−1.15, 0.57) *p =* 0.479	−0.63 (−2.64, 1.38) *p =* 0.514	0.90 (−2.11, 3.91) *p =* 0.535	−1.56 (−3.23, 0.11) *p =* 0.064	−1.26 (−2.53, 0.01) *p =* 0.052
Symptoms of Sleep Disorders Score (*1* vs. *0*) *	−3.55 (−4.13, −2.98) *p <* 0.001	−8.50 (−9.76, −7.25) *p <* 0.001	−2.69 (−5.93, 0.54) *p =* 0.096	−1.83 (−3.25, −0.41) *p =* 0.015	−2.03 (−2.82, −1.25) *p <* 0.001
Sleep Latency Score (*1* vs. *0*) *	−2.25 (−3.03, −1.47) *p <* 0.001	−5.50 (−7.74, −3.25) *p <* 0.001	−3.12 (−7.16, 0.92) *p =* 0.120	−1.20 (−2.96, 0.56) *p =* 0.167	−1.84 (−2.74, −0.94) *p <* 0.001
Daytime Sleepiness (Alertness) Score (*1* vs. *0*) *	−2.23 (−3.09, −1.36) *p <* 0.001	−5.77 (−7.42, −4.12) *p <* 0.001	−1.78 (−5.68, 2.13) *p =* 0.348	−0.37 (−1.58, 0.85) *p =* 0.527	0.50 (−0.86, 1.86) *p =* 0.442

* Multidimensional sleep health scores were computed by assigning a score of 0 or 1 based on self-reported sleep duration, sleep regularity, snoring/snorting, difficulty falling asleep, and daytime sleepiness (alertness). Participants received a score of 1 for sufficient sleep duration (≥7 h and <9 h), regular sleep schedules (weekday-weekend differences in sleep timing midpoint and sleep duration <2 h), never or rarely reporting snoring/snorting, having no difficulty falling asleep, and never or rarely reporting excessive daytime sleepiness. Otherwise, they were assigned a score of 0. Component scores were summed to create the multidimensional sleep health score ranging from 0–5, with higher scores representing more favorable sleep health. Those with scores of 0–1, 2–3, 4–5 were considered to have poor, moderate, and ideal sleep health respectively. ^†^ All models are adjusted for age, sex, race/ethnicity, education, marital status, and medication use.

**Table 3:** Multivariable-Adjusted Logistic Regression Models for Associations of Multidimensional Sleep Health with Odds of Cardiovascular Disease and Cardiometabolic Outcomes (n = 4555) ^||^.

Sleep Health Scores *	Obesity ^†^	Central Adiposity ^†^	Type 2 Diabetes^†^	Hypertension (Definition 1) ^‡^	Hypertension (Definition 2) ^‡^	Cardiovascular Disease ^§^
	OR (95%CI)	OR (95%CI)	OR (95%CI)	OR (95%CI)	OR (95%CI)	OR (95%CI)
Multidimensional Sleep Health Score *						
**Poor (0–1) (ref)**	1.00	1.00	1.00	1.00	1.00	1.00
***Moderate (2–3)***	0.44 (0.35, 0.57)	0.45 (0.33, 0.63)	0.90 (0.48, 1.67)	0.59 (0.42, 0.82)	0.47 (0.35, 0.64)	0.82 (0.56, 1.19)
***Ideal (4–5)***	0.27 (0.20, 0.37) *p-trend < 0.001*	0.32 (0.23, 0.45) *p-trend < 0.001*	0.55 (0.27, 1.09) *p-trend = 0.588*	0.38 (0.28, 0.52) *p-trend < 0.001*	0.26 (0.18, 0.36) *p-trend < 0.001*	0.57 (0.32–1.01) *p-trend = 0.625*
Sleep Duration Score (*1* vs. *0*) *	0.95 (0.80, 1.12)	0.99 (0.83, 1.18)	1.02 (0.74, 1.42)	0.90 (0.71, 1.12)	0.67 (0.53, 0.83)	0.66 (0.47, 0.92)
Sleep Regularity Score (*1* vs. *0*) *	1.06 (0.85, 1.33)	1.03 (0.88, 1.20)	1.34 (0.99, 1.81)	1.19 (0.93, 1.51)	1.24 (0.97, 1.60)	1.86 (1.37, 2.54)
Disordered Sleep Score (*1* vs. *0*) *	0.42 (0.35, 0.50)	0.45 (0.38, 0.54)	0.63 (0.48, 0.84)	0.60 (0.50, 0.72)	0.62 (0.49, 0.78)	1.01 (0.70, 1.45)
Sleep Latency Score (*1* vs. *0*) *	0.57 (0.47, 0.68)	0.68 (0.54, 0.87)	0.65 (0.45, 0.94)	0.53 (0.44, 0.65)	0.51 (0.41, 0.62)	0.59 (0.45, 0.78)
Daytime Sleepiness (Alertness) Score (*1* vs. *0*) *	0.57 (0.48, 0.67)	0.61 (0.51, 0.74)	0.54 (0.41, 0.71)	0.69 (0.55, 0.88)	0.56 (0.44, 0.70)	0.48 (0.35, 0.67)

* Multidimensional sleep health scores were computed by assigning a score of 0 or 1 based on self-reported sleep duration, sleep regularity, snoring/snorting, difficulty falling asleep, and daytime sleepiness (alertness). Participants received a score of 1 for sufficient sleep duration (≥7 h and <9 h), regular sleep schedules (weekday-weekend differences in sleep timing midpoint and sleep duration <2 h), never or rarely reporting snoring/snorting, having no difficulty falling asleep, and never or rarely reporting excessive daytime sleepiness. Otherwise, they were assigned a score of 0. Component scores were summed to create the multidimensional sleep health score ranging from 0–5, with higher scores representing more favorable sleep health. Those with scores of 0–1, 2–3, 4–5 were considered to have poor, moderate, and ideal sleep health respectively. ^†^ Obesity is defined as a BMI ≥ 30 kg/m^2^; central adiposity is defined as a waist circumference > 88 cm in women and > 102 cm in men; and type 2 diabetes is defined as fasting glucose ≥126 mg/dL or type 2 diabetes medication use. ^‡^ Hypertension definition 1 (2017 ACC/AHA definition): SBP/DBP ≥ 130/80 mmHg or hypertension medication use; hypertension definition 2 (JNC 7 definition): SBP/DBP ≥ 140/90 mmHg or hypertension medication use. ^§^ The CVD composite outcome includes heart failure, coronary heart disease, angina/angina pectoris, heart attack, or stroke. ^||^ All models are adjusted for age, sex, race/ethnicity, education, and marital status.

## Data Availability

NHANES data are available in a publicly accessible repository: https://www.cdc.gov/nchs/nhanes/index.htm (accessed on 15 July 2022).

## Abbreviations

AHA	American Heart Association
BMI	body mass index
BP	blood pressure
CMH	cardiometabolic health
CVD	cardiovascular disease
DBP	diastolic blood pressure
HTN	hypertension
NHANES	National Health and Nutrition Examination Survey
SBP	systolic blood pressure
T2D	type 2 diabetes
WC	waist circumference
